# Supplementary material for: Comorbidity patterns and sex differences in late-onset Alzheimer’s disease using electronic records
Source: iScience. 2026 Oct 8;29(11):117753. doi: 10.1016/j.isci.2026.117753 (PMC13677334; doi:10.1016/j.isci.2026.117753)
Supplement: Supplementary file 1 [file mmc1.pdf]

iScience, Volume *29*

Supplemental information

Comorbidity patterns and sex differences in late-onset Alzheimer's disease using electronic records

Yukari Katsuhara, Umair Khan, Zachary A. Miller, Isabel E. Allen, Tomiko T. Oskotsky, Marina Sirota, and Alice S. Tang

Supplemental Materials

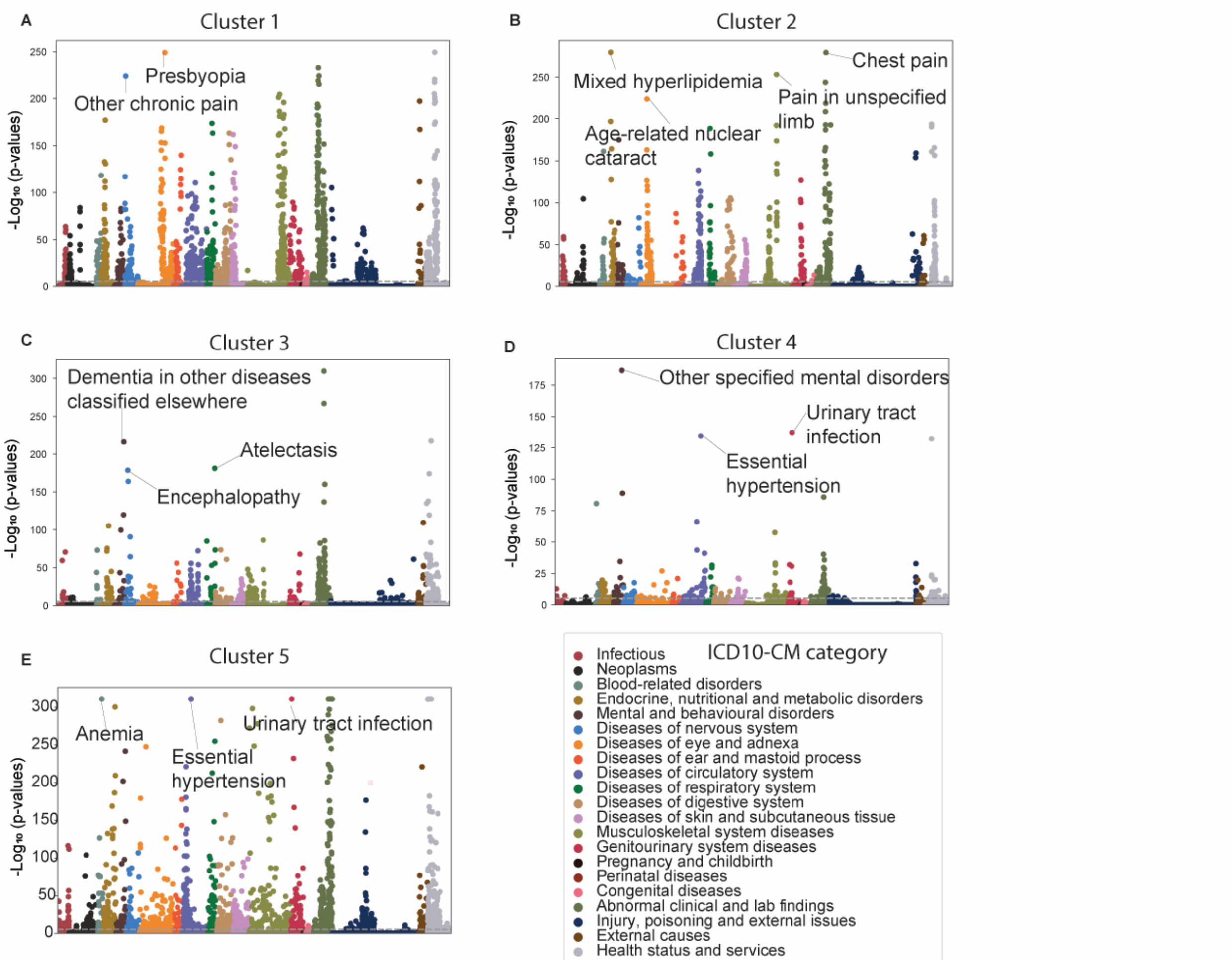

Supplemental Figure 1. Manhattan plots of cluster-specific comorbidity enrichment patterns. Manhattan plots displaying enriched ICD-10-CM codes identified using the two-sided Fisher's exact test (for cases < 5) or the Chi-squared test (for cases ≥ 5). Enrichment was determined based on a Bonferroni-corrected p-value < 0.05. ICD-10-CM, the International Classification of Diseases, 10th Revision, Clinical Modification. **A:** Cluster 1, **B:** Cluster 2, **C:** Cluster 3, **D:** Cluster 4, **E:** Cluster 5

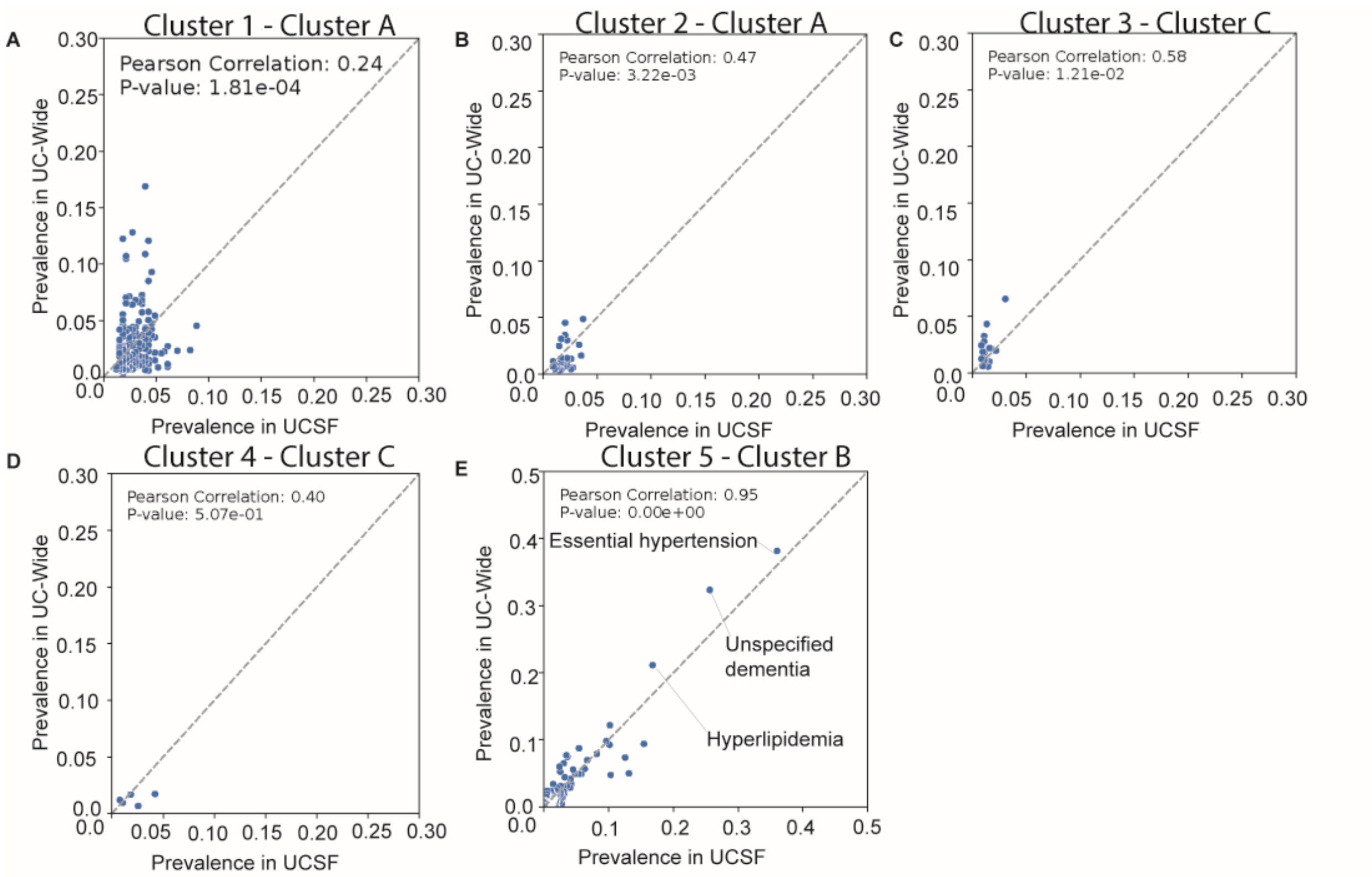

Supplemental Figure 2. Cross-dataset comparison of comorbidity prevalence. Comparison of comorbidity prevalence showing partial reproducibility of comorbidity patterns across the UCSF and UC-Wide datasets, particularly in Cluster 5.

A: Cluster 1 vs. Matched UC-Wide Cluster A, **B:** Cluster 2 vs. Matched UC-Wide Cluster A, **C:** Cluster 3 vs. Matched UC-Wide Cluster C, **D:** Cluster 4 vs. Matched UC-Wide Cluster C, **E:** Cluster 5 vs. Matched UC-Wide Cluster B

P-values were calculated from Pearson correlation analysis. UC, University of California.

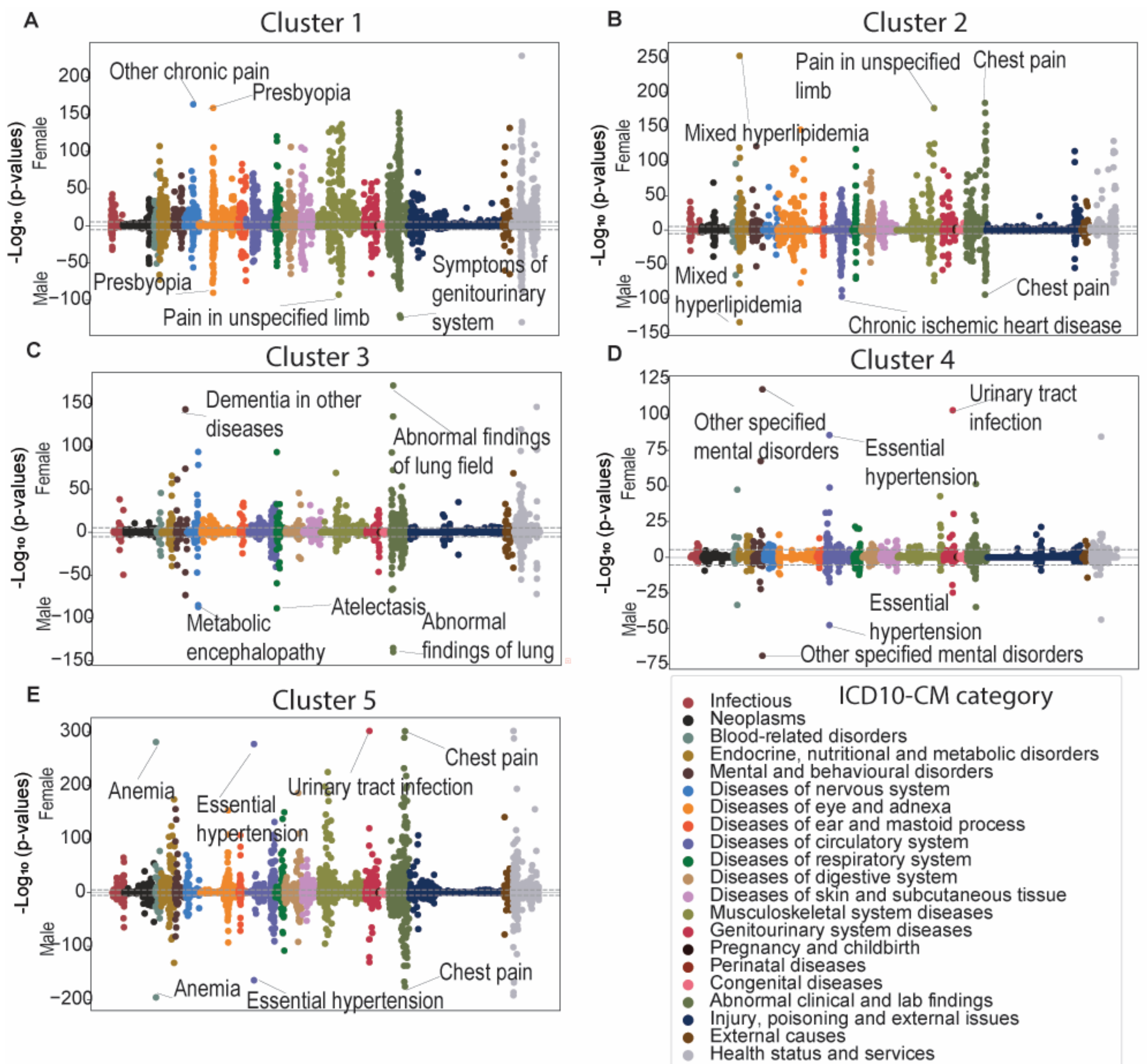

Supplemental Figure 3. Miami plots of sex-stratified comorbidity enrichment patterns across clusters.

Miami plots displaying enriched ICD-10-CM codes identified using the two-sided Fisher's exact test (for cases < 5) or the Chi-squared test (for cases ≥ 5). Enrichment was determined based on a Bonferroni-corrected p-value < 0.05. ICD-10-CM, the International Classification of Diseases, 10th Revision, Clinical Modification. **A:** Cluster 1, **B:** Cluster 2, **C:** Cluster 3, **D:** Cluster 4, **E:** Cluster 5

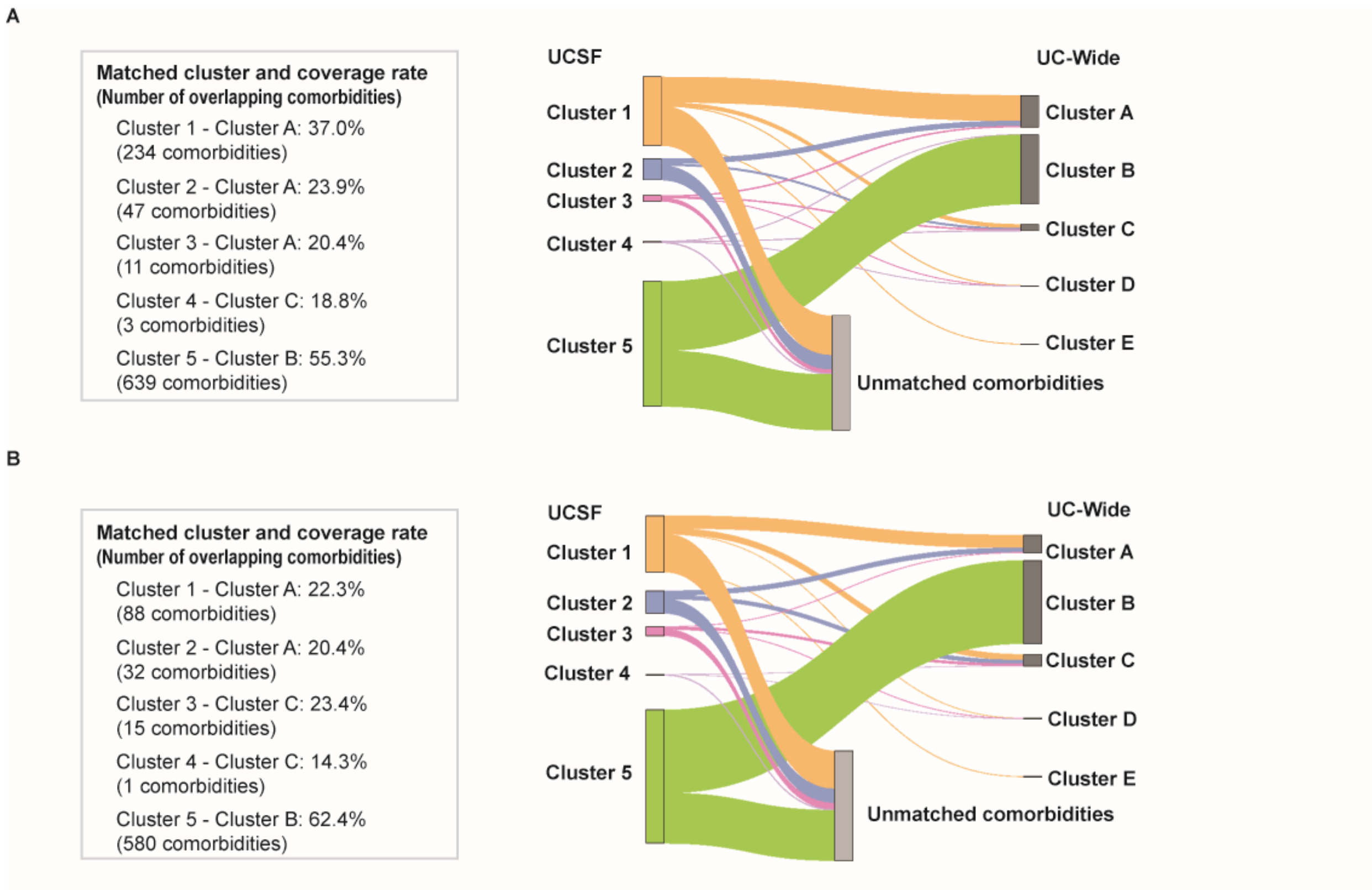

Supplemental Figure 4. Sex-stratified Sankey diagram and coverage rate

Sankey plot illustrating the partial overlap of significant comorbidities across UCSF and UC-Wide populations within each cluster. The coverage rate represents the proportion of UCSF cluster-specific comorbidities that remain significant in both the UCSF and UC-Wide datasets, identifying the most closely matched UC cluster. UC, University of California.

A: Female. Number of cluster-specific comorbidities: Cluster 1 (633), Cluster 2 (197), Cluster 3 (54), Cluster 4 (16), Cluster 5 (1,156), Cluster A (1,776), Cluster B (2,108), Cluster C (576), Cluster D (24), Cluster E (10)

B: Male. Number of cluster-specific comorbidities: Cluster 1 (395), Cluster 2 (157), Cluster 3 (64), Cluster 4 (7), Cluster 5 (929), Cluster A (1,053), Cluster B (1,660), Cluster C (604), Cluster D (33), Cluster E (13)

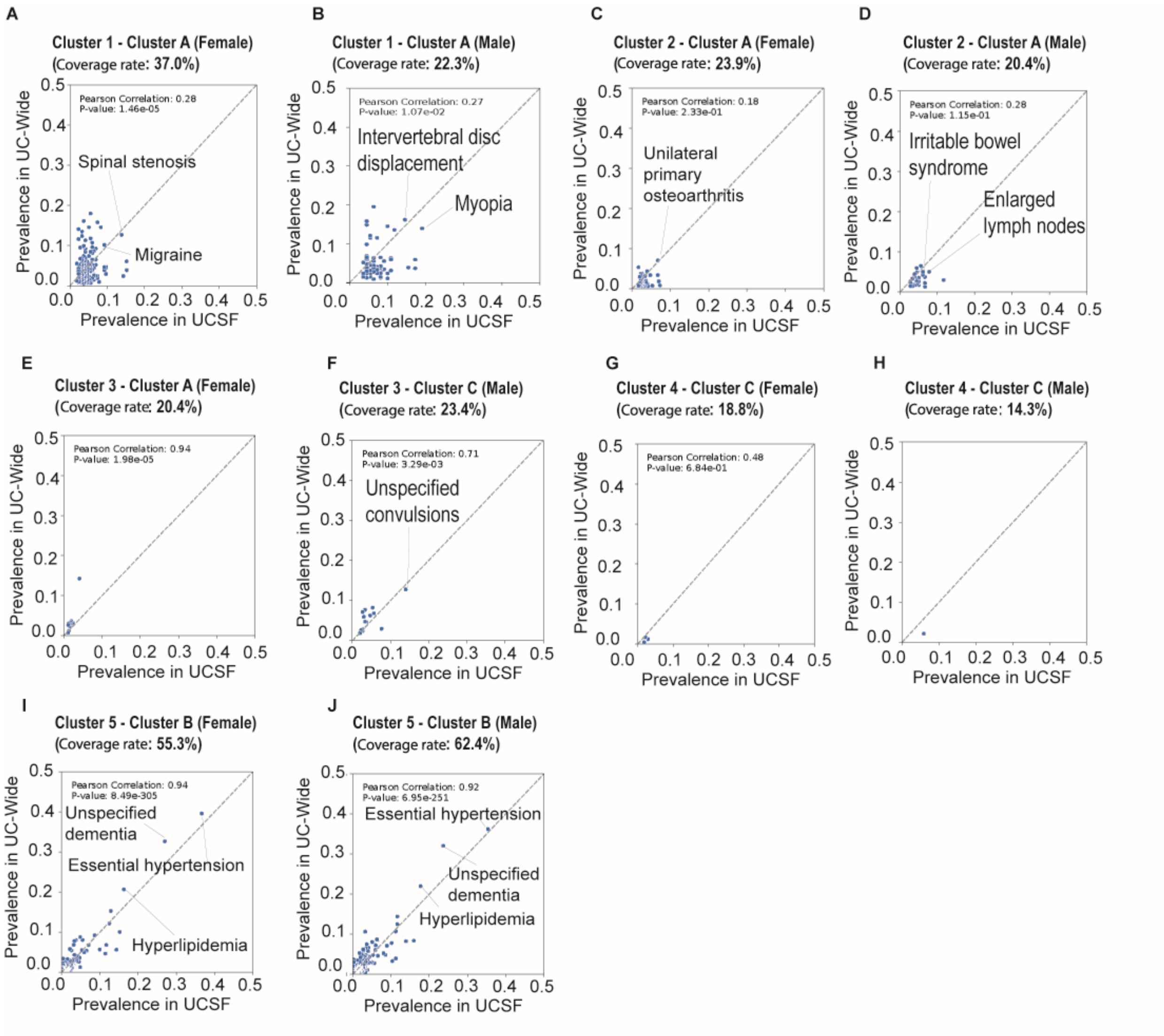

Supplemental Figure 5. Sex-stratified cross-dataset comparison of comorbidity prevalence.

Comparison of comorbidity prevalence showing partial reproducibility of comorbidity patterns across the UCSF and UC-Wide datasets, particularly for Clusters 1 and 5.

The covered rate represents the proportion of UCSF cluster-sex-specific comorbidities that remain significant in both the UCSF and UC-Wide datasets. **A, C, E, G, I**: Cluster 1–Cluster 5 (Female) vs. Matched UC-Wide Cluster (Female), **B, D, F, H, J:** Cluster 1–Cluster 5 (Male) vs. Matched UC-Wide Cluster (Male). P-values were calculated from Pearson correlation analysis. UC, University of California.

	UCSF								
Number of Cluster	WSS	ΔWSS (%)	Silhouette	ΔSilhouette (%)	Calinski Harabasz	ΔCH (%)	Davies Bouldin	ΔDB (%)	Smallest Cluster (%)
2	319553	–	0.439	-	497.64	–	3.7447	–	17.0% (n=1424)
3	312922	2.1%	0.284	35.3%	342.64	31.2%	4.6098	23.1%	8.8%(n=735)
4	308949	1.3%	0.272	4.2%	267.16	22.0%	4.8371	4.9%	4.8%(n=403)
5	306584	0.8%	0.159	41.5%	218.01	18.4%	5.1402	6.3%	3.9%(n=328)
6	305359	0.4%	0.019	88.1%	181.8	16.6%	5.6721	10.4%	3.9%(n=322)
	UC-Wide								
Number of Cluster	WSS	ΔWSS (%)	Silhouette	ΔSilhouette (%)	Calinski Harabasz	ΔCH (%)	Davies Bouldin	ΔDB (%)	Smallest Cluster (%)
2	1108599	-	0.273	-	1774.358	–	3.6563	–	27.0% (n=6996)
3	1089258	1.7%	0.141	48.4%	1132.773	36.2%	5.1086	39.7%	12.0% (n=3111)
4	1078519	1.0%	0.136	3.5%	848.604	25.1%	5.4058	5.8%	10.1% (n=2620)
5	1071218	0.7%	0.068	50.0%	683.732	19.4%	6.0116	11.2%	6.8% (n=1765)
6	1065004	0.6%	0.048	29.4%	581.301	15.0%	5.9059	-1.8%	3.7%(n=949)

Supplemental Table 1. Cluster performance metrics across clustering solutions

WSS, Within-cluster Sum of Squares; CH, Calinski Harabasz; DB, Davies Bouldin

4 Clusters

Cluster 1 (N=403)			Cluster 2 (N=1,293)			Cluster 3 (N=817)			Cluster 4 (N=5,850)		
ICD-10-CM		Prev.	ICD-10-CM		Prev.	ICD-10-CM		Prev.	ICD-10-CM		Prev.
I10	Z00.00	0.043	I10	Z78.9	0.098	F02.80	I10	0.068	I10	Z00.00	0.043
I10	Z23	0.043	I10	R07.9	0.093	F02.80	F03.90	0.066	I10	Z23	0.043
Z00.00	Z23	0.043	I10	N39.0	0.092	F03.90	I10	0.062	Z00.00	Z23	0.043
E78.5	I10	0.041	D64.9	I10	0.085	E78.5	F02.80	0.056	E78.5	I10	0.041
E78.5	Z00.00	0.039	F06.8	I10	0.084	E78.5	I10	0.053	E78.5	Z00.00	0.039
E78.5	Z23	0.039	E78.5	I10	0.082	F02.80	Z00.00	0.049	E78.5	Z23	0.039
I10	R05	0.039	F03.90	I10	0.080	E78.5	F03.90	0.048	I10	R05	0.039
I10	R07.9	0.038	I10	Z00.00	0.078	F02.80	Z71.89	0.048	I10	R07.9	0.038
R05	Z00.00	0.038	I10	Z23	0.073	F02.80	Z23	0.047	R05	Z00.00	0.038
R05	Z23	0.038	R53.81	R53.83	0.071	Z00.00	Z23	0.047	R05	Z23	0.038

5 Clusters

Cluster 1 (N=328)			Cluster 2 (N=538)			Cluster 3 (N=815)			Cluster 4 (N=1,521)			Cluster 5 (N=5,161)		
ICD-10-CM		Prev.	ICD-10-CM		Prev.	ICD-10-CM		Prev.	ICD-10-CM		Prev.	ICD-10-CM		Prev.
I10	Z00.00	0.035	I10	R07.9	0.053	F02.80	I10	0.068	I10	Z78.9	0.080	F02.80	I10	0.083
Z00.00	Z23	0.035	E78.5	I10	0.050	F02.80	F03.90	0.066	I10	N39.0	0.080	F02.80	F03.90	0.071
I10	Z23	0.035	I10	Z78.9	0.049	F03.90	I10	0.062	F03.90	I10	0.077	F03.90	I10	0.066
E78.5	I10	0.033	I10	Z00.00	0.048	E78.5	F02.80	0.056	F06.8	I10	0.068	E78.5	I10	0.062
E78.5	Z00.00	0.032	I10	N39.0	0.045	E78.5	I10	0.054	D64.9	I10	0.065	E78.5	F02.80	0.055
E78.5	Z23	0.032	D64.9	I10	0.045	F02.80	Z00.00	0.050	I10	R07.9	0.063	I10	R41.3	0.049
I10	R05	0.031	I10	Z23	0.045	E78.5	F03.90	0.048	E78.5	I10	0.058	F02.80	R41.3	0.047
R05	Z00.00	0.031	R53.81	R53.83	0.043	F02.80	Z23	0.048	F03.90	N39.0	0.055	F02.80	G31.84	0.045
F02.80	I10	0.031	I10	R53.81	0.043	Z00.00	Z23	0.048	F03.90	Z78.9	0.051	F02.80	Z66	0.042

Supplemental Table 2. Cluster-specific comorbidity pair prevalence for K = 4 and K = 5

Prev., Prevalence

ICD-10-CM, the International Classification of Diseases, 10th Revision, Clinical Modification.

Age in UCSF Cohort					Age in UC-Wide Cohort				
	Cluster 5	Cluster 1	Cluster 2	Cluster 3		Cluster A	Cluster B	Cluster C	Cluster D
Cluster 1	0.80	-	-	-	Cluster B	1	-	-	-
Cluster 2	0.00*	0.00*	-	-	Cluster C	0.00*	0.00*	-	-
Cluster 3	0.00*	0.00*	0.00*	-	Cluster D	0.00*	0.00*	0.00*	-
Cluster 4	0.00*	0.00*	0.38	0.00*	Cluster E	0.73	0.45	0.00*	0.00*
Number of comorbidities in UCSF Cohort					Number of comorbidities in UC-Wide Cohort				
	Cluster 5	Cluster 1	Cluster 2	Cluster 3		Cluster A	Cluster B	Cluster C	Cluster D
Cluster 1	0.00*	-	-	-	Cluster B	0.00*	-	-	-
Cluster 2	0.00*	0.00*	-	-	Cluster C	0.00*	0.00*	-	-
Cluster 3	0.00*	0.00*	0.10	-	Cluster D	0.00*	0.00*	0.00*	-
Cluster 4	0.00*	0.00*	0.00*	0.00*	Cluster E	0.00*	0.00*	0.00*	0.00*
Sex in UCSF Cohort					Sex in UC-Wide Cohort				
	Cluster 5	Cluster 1	Cluster 2	Cluster 3		Cluster A	Cluster B	Cluster C	Cluster D
Cluster 1	0.67	-	-	-	Cluster B	0.00*	-	-	-
Cluster 2	0.16	1	-	-	Cluster C	0.00*	0.00*	-	-
Cluster 3	1	1	1	-	Cluster D	0.00*	0.00*	1	-
Cluster 4	0.00*	0.94	0.49	0.00*	Cluster E	0.19	0.00*	0.00*	0.00*

Supplemental Table 3. Bonferroni-corrected p-values for pairwise comparisons across clusters

P-values for age and the number of comorbidities were calculated using the Kruskal-Wallis rank sum test with Bonferroni correction. P-values for pairwise comparisons of sex were obtained from proportion tests with Bonferroni correction. *, Adjusted p-value < 0.05; UC, University of California

	Cluster 1, *N* = 328 (Sex: Unknown N=1)				Cluster 2, *N* = 538 (Sex: Unknown N=0)				Cluster 3, *N* = 815 (Sex: Unknown N=0)			
Sex, N, *n* (%)	Female (N=217)		Male (N=110)		Female (N=358)		Male (N=180)		Female (N=514)		Male (N=301)	
Race, *n* (%)												
White or Caucasian	91	(42%)	57	(52%)	150	(42%)	98	(54%)	268	(52%)	171	(57%)
Other/Unknown	31	(14%)	13	(12%)	68	(19%)	30	(17%)	46	(8.9%)	29	(9.6%)
Asian	71	(33%)	37	(34%)	65	(18%)	25	(14%)	143	(28%)	79	(26%)
Black or African American	24	(11%)	3	(2.7%)	53	(15%)	17	(9.4%)	47	(9.1%)	18	(6.0%)
Native Hawaiian or Other Pacific Islander	0	0			22	(6.1%)	10	(5.6%)	10	(1.9%)	4	(1.3%)
Death Status, *n* (%)												
Alive	145	(67%)	73	(66%)	176	(49%)	82	(46%)	358	(70%)	192	(64%)
Deceased	72	(33%)	37	(34%)	182	(51%)	98	(54%)	156	(30%)	109	(36%)
Age (yr), Median (IQR)	90	(84-91)	89	(84-91)	90	(90-91	90	(90-91)	88	(82.3-90)	87	(81-90)
Comorbidities, Median (IQR)	313	(256-373)	314	(265.3-388.8)	129	(105-168)	138.5	(108.5-183)	112	(78.3-156.8)	118	(84-155)

	Cluster 4, *N* = 1,521 (Sex: Unknown N=0)				Cluster 5, *N* = 5,161 (Sex: Unknown N=18)			
Sex, *n* (%)	Female (N=1,083)		Male (N=438)		Female (N=3,143)		Male (N=2,000)	
Race, *n* (%)								
White or Caucasian	632	(58%)	265	(61%)	3,171	(60%)	1,400	(70%)
Other/Unknown	142	(13%)	63	(14%)	849	(16%)	307	(15%)
Asian	74	(6.8%)	35	(8.0%)	582	(11%)	118	(5.9%)
Black or African American	148	(14%)	32	(7.3%)	431	(8.1%)	81	(4.1%)
Native Hawaiian or Other Pacific Islander	87	(8.0%)	43	(9.8%)	282	(5.3%)	94	(4.7%)
Death Status, *n* (%)								
Alive	635	(59%)	277	(63%)	3,959	(74%)	1,681	(84%)
Deceased	448	(41%)	161	(37%)	1,356	(26%)	319	(16%)
Age (yr), Median (IQR)	91	(90-91)	90	(90-91)	90	(82-91)	88	(80-91)
Comorbidities, Median (IQR)	46	(32-64)	49	(35-66.8)	12	(5-21)	12	(5-22)

Supplemental Table 4. Sex-stratified patient demographics in UCSF. Medians and interquartile ranges are presented as median (25th percentile-75th percentile). "Comorbidities" refers to "Number of comorbidities," which represents the total count of diagnoses per patient. The category "Unknown" in Sex includes records classified as “Unknown.” The category "Other/Unknown" in Race includes records classified as “American Indian or Alaska Native,” “Other,” “0,” “Unknown/Declined,” “Unknown,” and “Declined.”
