# Supplementary material for: Comorbidity patterns and sex differences in late-onset Alzheimer’s disease using electronic records
Source: iScience. 2026 Oct 8;29(11):117753. doi: 10.1016/j.isci.2026.117753 (PMC13677334; doi:10.1016/j.isci.2026.117753)
Supplement: Supplementary file 2 [file mmc4.pdf]

STROBE Statement—checklist of items that should be included in reports of observational studies

	Item No.	Recommendation	Page No.	Relevant text from manuscript
**Title and abstract**	1	(*a*) Indicate the study's design with a commonly used term in the title or the abstract	*Summary (page 2)*	*"In this retrospective study, we applied unsupervised learning techniques to electronic medical records from UCSF to characterize Alzheimer's disease comorbidity patterns."*
		(*b*) Provide in the abstract an informative and balanced summary of what was done and what was found	*Summary (page 2)*	*"In this retrospective study, we applied unsupervised learning techniques to electronic medical records from UCSF to characterize Alzheimer's disease comorbidity patterns." "Our study provides a structured characterization of comorbidity heterogeneity in AD and highlights sex-related differences, with findings interpreted as descriptive and hypothesis-generating."*
**Introduction**				
Background/rationale	2	Explain the scientific background and rationale for the investigation being reported	*Introduction (page 3)*	*"Alzheimer's disease (AD) is the most common cause of dementia. With rising prevalence and no curative treatments, AD represents a major public health crisis worldwide…"*
Objectives	3	State specific objectives, including any prespecified hypotheses	*Introduction (page 4)*	*"In this study, we apply unsupervised clustering to a large EMR dataset of AD patients, consisting largely of LOAD presentations, to investigate heterogeneity within the AD population rather than relying on traditional case-control comparisons. By leveraging real-world clinical data, we aim to characterize comorbidity patterns and examine potential sex-specific differences. "*
**Methods**				
Study design	4	Present key elements of study design early in the paper	*Methods (page 32)*	*"This is a retrospective cross-sectional study using EMR data. "*
Setting	5	Describe the setting, locations, and relevant dates, including periods of recruitment, exposure, follow-up, and data collection	*Methods (page 32)*	*"All clinical data used in this study were obtained from the University of California's Health Data Warehouse (UCHDW) and the University of California Data Discovery Platform (UCDDP)…"*
Participants	6	(*a*) *Cross-sectional study*—Give the eligibility criteria, and the sources and methods of selection of participants	*Methods (page 33)*	*"Patients were included in the study if they met the inclusion criteria of an estimated age above 64 years and a diagnosis of AD based on the presence of the ICD-10-CM codes G30.1, G30.8, or G30.9. …Only patients with at least one remaining documented*

				comorbidity in addition to AD were included in the analysis. "
		(*b*) *Cohort study*—For matched studies, give matching criteria and number of exposed and unexposed<br>*Case-control study*—For matched studies, give matching criteria and the number of controls per case	*NA*	*NA*
Variables	7	Clearly define all outcomes, exposures, predictors, potential confounders, and effect modifiers. Give diagnostic criteria, if applicable	*Methods (page 35)*	*"For each diagnosis, odds ratios, 95% confidence intervals, and p-values were calculated based on 2×2 contingency tables."*<br>*"Patients were included in the study if they met the inclusion criteria of an estimated age above 64 years and a diagnosis of AD based on the presence of the ICD-10-CM codes G30.1, G30.8, or G30.9…"*
Data sources/ measurement	8*	For each variable of interest, give sources of data and details of methods of assessment (measurement). Describe comparability of assessment methods if there is more than one group	*Methods (page 32)*	*"All clinical data used in this study were obtained from the University of California's Health Data Warehouse (UCHDW) and the University of California Data Discovery Platform (UCDDP)…"*
Bias	9	Describe any efforts to address potential sources of bias	*Limitations (page 28)*	*"EMRs are subject to potential misclassification, missing data, and variability in coding practices. AD cases were identified based on diagnosis codes without biomarker, imaging, or neuropathological confirmation. … These approaches may potentially limit generalizability and introduce selection bias toward patients with higher medical complexity. ….Furthermore, our analysis was limited to medical institutions in California, necessitating caution when extrapolating these findings to other regions or populations."*
Study size	10	Explain how the study size was arrived at	*Results (page 33)*	*"Patients were included in the study if they met the inclusion criteria of an estimated age above 64 years and a diagnosis of AD based on the presence of the ICD-10-CM codes G30.1, G30.8, or G30.9. …Only patients with at least one remaining documented comorbidity in addition to AD were included in the analysis. ... Among 8,804 patients who otherwise met the AD eligibility criteria, 441 patients (5.0%) had no recorded comorbidity beyond AD and were excluded, resulting in a final analytic cohort of 8,363 AD patients. "*

Continued on next page

Quantitative variables	11	Explain how quantitative variables were handled in the analyses. If applicable, describe which groupings were chosen and why	*Methods (page 35)*	*"For each diagnosis, odds ratios, 95% confidence intervals, and p-values were calculated based on 2×2 contingency tables. The directionality of associations was determined based on the odds ratios. In addition to statistical significance, we calculated the prevalence of each comorbidity within each cluster to contextualize clinical relevance. Prevalence was defined as the proportion of patients within a given cluster with a specific diagnosis. "*
Statistical methods	12	(*a*) Describe all statistical methods, including those used to control for confounding	*Methods (page 35)*	*"To examine overall trends within each cluster, we performed enrichment analyses by comparing the frequency of each comorbidity in a given cluster with that in all other clusters combined…. For each diagnosis, the proportion of patients in the target cluster was compared to the proportion in the other clusters using Fisher's exact test when the sample size was below five or the chi-squared test when the sample size was five or more. Statistical significance was determined using a Bonferroni-corrected threshold of p-value < 0.05. ...In addition to statistical significance, we calculated the prevalence of each comorbidity within each cluster to contextualize clinical relevance. ...For interpretation of cluster characteristics, emphasis was placed on diagnoses with prevalence ≥5%, whereas lower-prevalence diagnoses were interpreted cautiously as exploratory signals. ..The statistical significance of associations and effect sizes of associations were visualized using volcano plots, while Manhattan plots (for the overall population) and Miami plots (for sex-stratified populations) illustrated the distribution of p-values across diagnoses."*
		(*b*) Describe any methods used to examine subgroups and interactions	*Methods (page 34)*	*"Our study examined both males and females using stratified analyses to identify sex differences in AD data-driven groupings. By conducting these analyses, we aimed to clarify potential sex-specific variations in disease characteristics."*
		(*c*) Explain how missing data were addressed	*Methods (page 34)*<br><br>*Limitations (page 28)*	*"Missing diagnoses were treated as absence of documentation and encoded as zeros in the binary diagnosis matrix. No imputation procedures were applied."*<br>*"Third, the use of EMRs introduces several limitations. EMRs are subject to potential misclassification, missing data, and variability in coding practices. "*

		(*d*) *Cross-sectional study*—If applicable, describe analytical methods taking account of sampling strategy		*NA*
		(*e*) Describe any sensitivity analyses	*Methods (page 36)*	*"To evaluate cross-dataset consistency of the clustering and comorbidity enrichment results, the same approach used for the UCSF dataset was applied to the UC-Wide dataset…"*
**Results**				
Participants	13*	(a) Report numbers of individuals at each stage of study—eg numbers potentially eligible, examined for eligibility, confirmed eligible, included in the study, completing follow-up, and analysed	*Results (page 5)*	*"We identified 8,804 patients who met the AD case definition in the UCSF EMRs. Of these, 441 patients (5.0%) had no recorded comorbidities beyond AD and were excluded, resulting in a final cohort of 8,363 AD patients (5,315 female (64%); median age: 90.0 [IQR: 84.0–91.0]) (Figure 1). "*
		(b) Give reasons for non-participation at each stage	*Results (page 5)*	*"We identified 8,804 patients who met the AD case definition in the UCSF EMRs. Of these, 441 patients (5.0%) had no recorded comorbidities beyond AD and were excluded, resulting in a final cohort of 8,363 AD patients (5,315 female (64%); median age: 90.0 [IQR: 84.0–91.0]) (Figure 1). "*
		(c) Consider use of a flow diagram	*Page 6*	*Figure 1*
Descriptive data	14*	(a) Give characteristics of study participants (eg demographic, clinical, social) and information on exposures and potential confounders	*Results (page 5)*	*"We identified 8,363 AD patients from the UCSF EMRs (5,315 female (64%); median age: 90.0 [IQR: 84.0–91.0]) (Figure 1). "*
		(b) Indicate number of participants with missing data for each variable of interest	*Document S1.*	*Supplemental Table 4. (Sex: Unknown: XX)*
		(c) *Cohort study*—Summarise follow-up time (eg, average and total amount)	*NA*	*NA*
Outcome data	15*	*Cohort study*—Report numbers of outcome events or summary measures over time	*NA*	*NA*
		Case-control study—Report numbers in each exposure category, or summary	*NA*	*NA*

		measures of exposure		
		Cross-sectional study—Report numbers of outcome events or summary measures	*Results (page 7)*	*“The analysis data consisted of patient-level diagnostic records, where each row represented an individual patient and each column corresponded to one of 33,031 unique diagnosis names across 8,363 AD patients…”*
Main results	16	(*a*) Give unadjusted estimates and, if applicable, confounder-adjusted estimates and their precision (eg, 95% confidence interval). Make clear which confounders were adjusted for and why they were included	*Data S1*	*Data S1*
		(*b*) Report category boundaries when continuous variables were categorized	*NA*	*NA*
		(*c*) If relevant, consider translating estimates of relative risk into absolute risk for a meaningful time period	*NA*	*NA*

Continued on next page

Other analyses	17	Report other analyses done—eg analyses of subgroups and interactions, and sensitivity analyses	*Results (pages 15 and 18),*	*"To further evaluate the consistency of comorbidity enrichment across datasets, we examined the correlation of shared comorbidities using Log-Log plots (Figure 7B) …"* *"After illustrating the distribution of statistical significance in Miami plots, we identified cluster-sex-specific comorbidities using UpSet plots (Figure 4, Figure S3)."*
**Discussion**				
Key results	18	Summarise key results with reference to study objectives	*Discussion (page 23)*	*"Enrichment analysis revealed clinical heterogeneity, and sex-stratified analysis highlighted potential sex-specific differences…"*
Limitations	19	Discuss limitations of the study, taking into account sources of potential bias or imprecision. Discuss both direction and magnitude of any potential bias	*Limitations (page 27 and 28)*	*"There are several limitations in this study. First, the presence of imbalanced data resulted in some odds ratios being disproportionately inflated…"* *"Third, the use of EMRs introduces several limitations. EMRs are subject to potential misclassification, missing data, and variability in coding practices. AD cases were identified based on diagnosis codes without biomarker, imaging, or neuropathological confirmation. … These approaches may potentially limit generalizability and introduce selection bias toward patients with higher medical complexity. Due to the deidentification process, age data were truncated in both datasets. In the UCSF dataset, birth dates prior to 1930 were recoded to 1930, whereas individuals aged 91 years or older were assigned an age of 91 years in the UC-Wide dataset. …Furthermore, our analysis was limited to medical institutions in California, necessitating caution when extrapolating these findings to other regions or populations."*
Interpretation	20	Give a cautious overall interpretation of results considering objectives, limitations, multiplicity of analyses, results from similar studies, and other relevant evidence	*Discussion (page 26)*	*"Overall, while these EMR-derived comorbidity profiles provided meaningful insights into patterns of co-occurring conditions and the heterogeneity of AD, we acknowledge that administrative codes alone cannot fully capture the biological complexity in AD. Because AD and comorbidity diagnoses were based on administrative codes without biomarker or imaging confirmation, the study population may have included patients with mixed dementia, non-Alzheimer dementias, or miscoded diagnoses."*
Generalisability	21	Discuss the generalisability (external validity) of the study results	*Discussion (page 23)*	*"While clusters characterized by either high overall comorbidity burden or relatively low burden showed some consistency across datasets, more cluster-*

				specific or lower-prevalence signals showed limited reproducibility, suggesting limited generalizability of finer-grained patterns."
**Other information**				
Funding	22	Give the source of funding and the role of the funders for the present study and, if applicable, for the original study on which the present article is based	*Acknowledgements (page 30)*	*"This research was primarily supported by grants from the National Institute on Aging (NIA) (R01AG060393 and R01AG057683)."*

*Give information separately for cases and controls in case-control studies and, if applicable, for exposed and unexposed groups in cohort and cross-sectional studies.

Note: An Explanation and Elaboration article discusses each checklist item and gives methodological background and published examples of transparent reporting. The STROBE checklist is best used in conjunction with this article (freely available on the Web sites of PLoS Medicine at http://www.plosmedicine.org/, Annals of Internal Medicine at http://www.annals.org/, and Epidemiology at http://www.epidem.com/). Information on the STROBE Initiative is available at www.strobe-statement.org.
